# Development and psychometric validation of the soft skills scale for primary school students

**DOI:** 10.3389/fpsyg.2026.1906409

**Published:** 2026-08-11

**Authors:** Hanife Esen Aygün, Mehmet Aşıkcan, Şerife Gonca Zeren

**Affiliations:** 1 Department of Primary School Education, Canakkale Onsekiz Mart University, Canakkale, Türkiye; 2 Department of Primary School Education, Necmettin Erbakan University, Konya, Türkiye; 3 Department of Guidance and Psychological Counseling, Canakkale Onsekiz Mart University, Canakkale, Türkiye

**Keywords:** development scale, primary school students, psychometric validation, school psychology, soft skills

## Abstract

This study aimed to develop a Soft Skills Scale for primary school students and to determine its psychometric properties. A total of 2,418 primary school students participated in the scale development process. Principal component analysis revealed a six-factor, 19-item structure explaining 41.37% of the total variance. Confirmatory factor analysis provided additional evidence supporting the proposed six-factor structure, with acceptable model fit indices. Criterion-related validity was supported by a moderate positive correlation. Test-retest reliability, assessed over a four-week period, yielded a correlation coefficient of 0.83, indicating strong temporal stability. Internal consistency coefficients are Cronbach's alpha of 0.79 and McDonald's omega of 0.80 for the overall scale. The findings provide evidence supporting the reliability and construct validity of the SSS-S for assessing soft skills among primary school students within the context of the current sample. The Soft Skills Scale for primary school students address a significant gap in literature by providing one of the first scale designed to comprehensively assess interpersonal, cognitive, emotional, and behavioral components of soft skills in primary school students. The developed scale is expected to contribute to classroom-based interventions and school counseling practices. Additionally, the SSS-S has the potential to contribute to future international comparative studies. However, additional evidence regarding cross-cultural validity and measurement invariance is needed before such applications can be supported.

## 1 Introduction

In today's understanding of child development, supporting children's soft skills is gaining increasing importance, as these skills play a vital role in fostering social, emotional, behavioral, and cognitive development from an early age. Soft skills enable individuals to effectively navigate everyday situations, such as building social relationships, managing emotions, taking responsibility, and solving problems (Alt et al., 2023; Van den Beuken et al., 2025).

Researchers have not reached a consensus on what constitutes soft skills (Pinos Ullauri et al., 2024; Touloumakos, 2020). These skills refer to self-regulatory competencies that facilitate effective interpersonal interaction, in contrast to cognitive or hard skills, which involve the ability to perform well in standardized academic tasks (Farrington et al., 2012; Tseng et al., 2019). The term “soft skills” is often used interchangeably with other concepts such as future skills (World Economic Forum, 2020) 21st century skills (Partnership for 21st Century Learning, 2009), social-emotional skills (Collaborative for Academic, Social, and Emotional Learning, 2013), transversal skills (United Nations Educational, Scientific and Cultural Organization, 2014), life skills (World Health Organization, 1994), generic competence (The Tuning Project, 2021). Such terminological variations have emerged due to translation differences across languages (Cinque, 2016). While these concepts partially overlap with soft skills, they also diverge in some aspects (see in Table 1).

**Table 1:** Conceptual and theoretical models associated with soft skills.

Concepts/framework	References	Aim	Scope/components	Relation with soft skills
Personality traits	Goldberg, 1992; Costa and McCrae, 1992 (Big Five)	Identifying enduring tendencies and character structures of individuals	Extraversion, conscientiousness, openness, agreeableness, emotional stability	It overlaps with some soft skills; however, it primarily describes individual tendencies.
Life skills	World Health Organization, 1994	Developing individuals' coping skills for dealing with life.	Decision making, problem solving, creative/critical thinking, communication, interpersonal skills, and emotion management	They involve the fundamental building blocks of soft skills.
Emotional Intelligence (EI)	Goleman, 1995 Mayer and Salovey, 1997	Developing the skills to recognize, manage, and use emotions.	Self-awareness, self-regulation, emotional empathy, and social skills.	It lies at the heart of soft skills in their emotional dimension.
21st century skills	Partnership for 21st Century Learning, 2009	Preparing students for 21st-century work and life skills	Learning and innovation skills, information-media-technology skills, and life and career skills	Soft skills are related to the life and career skills component of this framework
Social-emotional skills	Collaborative for Academic, Social, and Emotional Learning, 2013	To support social and emotional development.	Self-awareness, self-management, social awareness, relationships, and responsible decision-making	They reflect the emotional and social dimensions of soft skills.
Transversal skills	United Nations Educational, Scientific and Cultural Organization, 2014	Global citizenship and readiness for lifelong learning	Critical thinking, problem solving, creativity, communication, collaboration, and cultural awareness	It approaches soft skills from a more universal perspective.
Competence for democratic culture	Council of Europe, 2023	Raising effective and responsible individuals in a democratic society	Critical thinking, respect, participation, compromise, and openness to pluralism	It supports the societal dimensions of soft skills
Core SEL competencies	Organisation for Economic Co-operation and Development, 2015	Integrating social and emotional learning into policies	Self-awareness, self-regulation, collaboration, empathy, and responsibility	It supports the development of soft skills at the policy level
Character education/virtues	Jubilee Centre for Character and Virtues, 2017	Fostering moral character and virtuous individuals	Empathy, honesty, responsibility, respect, and perseverance	It aligns with ethical and value-based soft skills
Global competence	Organisation for Economic Co-operation and Development, 2018	Developing intercultural interaction and sensitivity to global issues	Cultural awareness, empathy, open-mindedness, and taking action	It encompasses soft skills components such as empathy, communication, and collaboration
Future skills/skills for tomorrow	World Economic Forum, 2020	Preparation for the future world of work and its uncertainties.	Creativity, flexibility, emotional resilience, collaboration, and openness to learning	Particularly dimensions such as resilience and flexibility reflect the modern framework of soft skills
Generic competences	The Tuning Project, 2021	Defining general competences within the European higher education area	Teamwork, communication, adaptability, ethical responsibility, and leadership	It overlaps in the context of interpersonal relations and cognitive skills; particularly defined at the higher education level

The comparative framework presented in Table 1 reveals that the concept of soft skills encompasses not only individual attributes but also social, emotional, behavioral, cognitive, cultural, and moral dimensions, highlighting its multidimensional nature. This eclectic structure of soft skills that shaped by the perspectives of various disciplines offers diverse approaches for implementation in education, including assessment, curriculum development, and intervention design.

Although some researchers define soft skills more narrowly as non-academic personality traits (Alex, 2009), current literature widely defines these skills as a broad set of humanistic, cognitive, and social, emotional, behavioral skills (Chernyshenko et al., 2018; Schoon, 2021; Soto et al., 2022). For example, Caggiano et al. (2020) classified soft skills into four dimensions: intrapersonal, interpersonal, activity development, and impression management. Similarly, De Campos et al. (2020) categorize it into six categories; problem solving and critical thinking, communication, teamwork, ethical perspective, emotional intelligence, creative thinking. In another classification, Succi and Canovi (2020) grouped soft skills into three categories: personal, social, and methodological skills. Educational research on soft skills often adopts a three-dimensional framework comprising interpersonal, cognitive, and emotional components (Kyllonen, 2013; Luo and Li, 2025; Orih et al., 2024). Taking together, these perspectives suggest that soft skills constitute a comprehensive construct encompassing how individuals think, feel, behave, and interact with others across various life contexts.

Accordingly, this study conceptualizes soft skills as a multidimensional set of learnable competencies that enable primary school students to regulate their thoughts, emotions, and behaviors, interact effectively with others, and respond adaptively to everyday academic and social situations (Esen-Aygün et al., 2026). This conceptualization draws on the common elements shared across major international frameworks, which consistently emphasize interpersonal, cognitive, and emotional competencies despite differences in terminology. Based on this synthesis and the developmental characteristics of primary school children, the present study operationalizes soft skills through three interrelated domains: interpersonal skills, emotional control, and cognitive skills (Guerra-Báez, 2019). These domains provide the conceptual foundation for the development and validation of the proposed measurement instrument.

Although many high-quality studies have investigated the characteristics of soft skills in adults (Byrne et al., 2020; Tadjer et al., 2020; Traylor et al., 2022) and adolescents (Devedzic et al., 2018; Escolà-Gascón and Gallifa, 2022; Heckman and Kautz, 2012) there is still a lack of standardized measurement instruments capable of accurately assessing primary school students' soft skills. This may be due to the inherent difficulty of measuring soft skills. Moreover, the fact that the concept of soft skills is often associated with employability may also have contributed to this situation (Bhaskar et al., 2025; Haidar, 2025; Romanenko et al., 2024). Nevertheless, the lack of a psychometrically validated instrument to measure the acquisition and development of these skills among primary school students indicates a significant gap in literature. To address this gap the current study aimed to develop a soft skills scale for primary school students and to determine its psychometric properties.

### 1.1 Current study

Soft skills reflect children's internal attributes the cultural context and social environment play a prominent role in shaping these skills (Krasnoshchok et al., 2024). Therefore, the development of culturally situated assessment tools is essential for accurately capturing the context-specific dynamics of soft skills and generating meaningful evidence. Notably, the concept of soft skills has not yet achieved conceptual clarity within the Turkish educational context (Hevedanli, 2025). In this study, the comprehensive educational taxonomy of soft skills encompassing interpersonal skills, emotional control, and cognitive skills was adopted as theoretical foundation for scale development. Although prior studies have developed instruments targeting soft skills in adolescent (Escolà-Gascón and Gallifa, 2022) and higher education populations (Jardim et al., 2022), to the best of our knowledge, no psychometrically validated tool has comprehensively assessed the interpersonal, emotional, and cognitive dimensions of soft skills among primary school students. Unlike existing instruments that tend to assess social-emotional competencies or academic-related skills (Gresham and Elliott, 1990; Lucisano and du Mérac, 2019; Merrell et al., 2011; Organisation for Economic Co-operation and Development, 2021; Soto et al., 2022), the SSS-S offers an integrative framework within a developmentally appropriate structure for primary school students. Thus, the SSS-S addresses this gap by integrating social, emotional, behavioral, and cognitive skills within a unified framework tailored to primary school students.

## 2 Materials and methods

### 2.1 Development process of scale

The Soft Skills Scale for Primary School Students (SSS-S) was developed within the scope of this study. In this process, the stages of *item development, content validity, and piloting* were followed.

#### 2.1.1 Item development

The item development process began with a comprehensive literature review on soft skills and related conceptual frameworks. Attention was paid to identifying competencies consistently emphasized in major international frameworks and considered developmentally appropriate for primary school students. In line with the conceptual framework adopted in this study, the initial item pool was developed to represent the three dimensions of soft skills: interpersonal skills, emotional control, and cognitive skills. These three dimensions were selected because they represent the competencies most consistently identified across contemporary educational frameworks and are considered developmentally appropriate and observable in primary school children. Based on this conceptual framework, the research team created an initial pool of 75 items. The initial item pool was intentionally developed to provide balanced coverage across the three conceptual domains. Each item was written to reflect observable behaviors that primary school students might exhibit in daily academic and social contexts. To maximize comprehensibility, word choice was kept simple and age-appropriate for primary school students. Following an internal review by the research team, the preliminary item pool was examined for conceptual appropriateness, redundancy, developmental appropriateness, and consistency with the operational definition of soft skills adopted in this study. Items demonstrating conceptual overlap, limited alignment with the target construct, or insufficient developmental appropriateness were removed. This refinement process resulted in a preliminary version of the scale. The content validity of these items is assessed in the following section. The final preliminary version of the SSS-S consisted of 28 items, including five reverse-worded items, and employed a three-point Likert-type response format ranging from “Always” to “Sometimes” and “Never.”

#### 2.1.2 Content validity

To establish content validity, the initial item pool was independently reviewed by three experts with expertise in educational sciences, educational measurement, and primary education. The experts evaluated the items regarding their relevance to the construct, clarity of wording, comprehensibility, and age appropriateness. A formal CVI was not calculated because the expert panel consisted of only three experts. Instead, unanimous expert agreement was adopted as the decision criterion. Based on the experts' qualitative feedback, items with unclear wording or limited relevance to the target construct were revised. The revised version of the scale was finalized after incorporating the experts' recommendations. After the expert opinions, three students who had similar characteristics to the target group were asked to evaluate the questions in terms of meaning and clarity. Following minor wording revisions based on students' feedback, the preliminary item pool was considered ready for pilot testing.

#### 2.1.3 Piloting

The 28-item measurement tool was applied to a class of thirty-eight students for piloting purposes. However, the students had difficulty understanding and answering the reverse items. For this reason, five reverse items written during the item pool studies of the measurement tool were removed from the item pool. The scale was once again submitted to expert review, and after expert consensus, its initial version consisting of twenty-three items was ready for testing. The data obtained from these students were not included in the principal component analysis (PCA) data set.

### 2.2 Psychometric evaluation of scale

**Figure 1:**
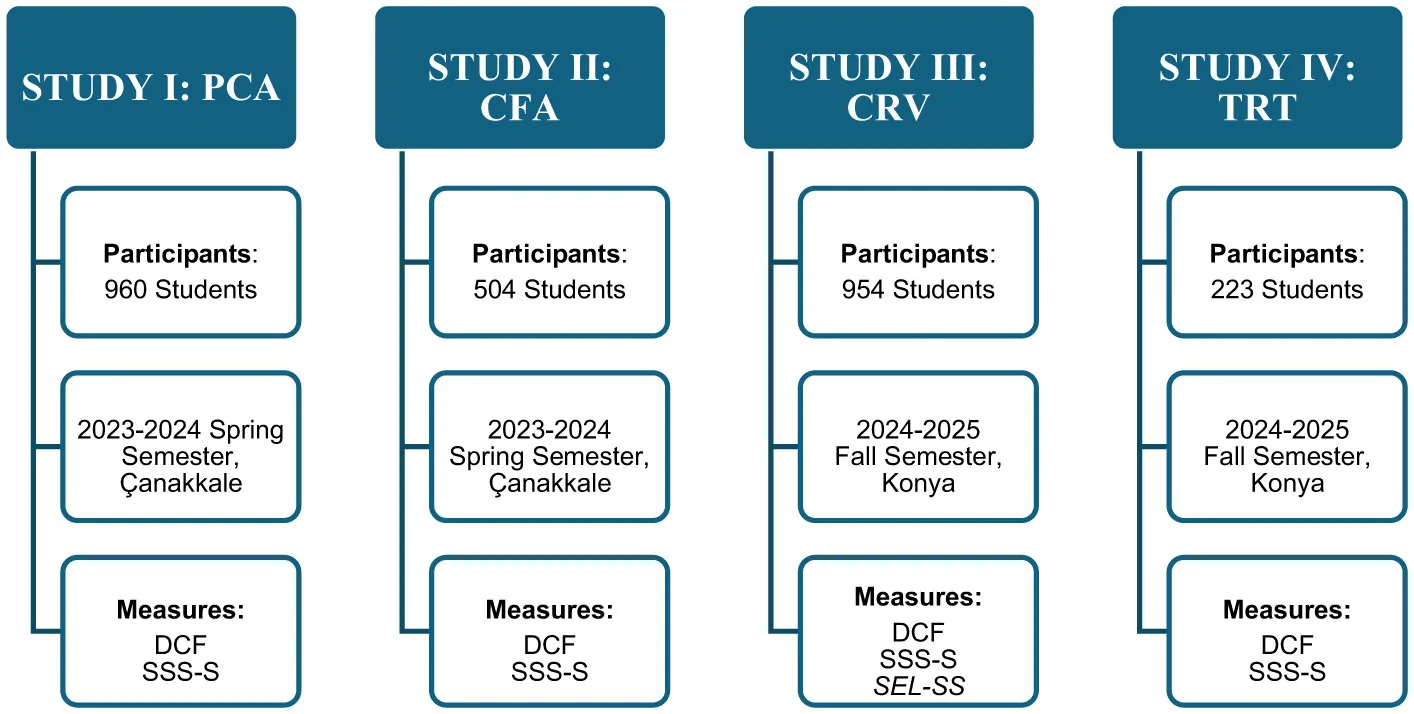
Studies for scale development procedures.

We conducted four separate studies to develop the Soft Skills Scale for primary school students and to determine its psychometric properties. First, in Study I, we conducted a PCA to determine the structure of the scale. Then, in Study II, we performed a confirmatory factor analysis (CFA) to examine whether this structure was confirmed as a model. In Study III, we tested the criterion-related validity (CRV) of the scale by comparing it with similar scales in the literature. Finally, in Study IV, we conducted a test–retest reliability (TRT) analysis. The procedures followed in the studies, the tests administered, and the number of participants is shown in Figure 1.

As shown in Figure 1, a total of 2,418 students from two different cities participated in the study. The students who took part in Study IV were a subgroup of those in Study III; therefore, they were not included in the total count. In Study I, the initial 23-item version of the SSS-S was administered, while in Studies II, III, and IV, the final version was used.

### 2.3 Participants

Prior to the application of the scale, ethical and legal permissions were obtained. Subsequently, parental informed consent forms were sent to the parents of the children through the teachers. Students who returned the parental informed consent forms were included in the study. Participants were determined through simple random sampling. Inclusion criteria for participation were being enrolled in the target grade level, obtaining parental consent, and voluntarily agreeing to participate in the study. Students who did not provide complete responses to the scale or who were absent during data collection were excluded from the analyses. PCA and CFA were performed on data collected from separate student groups approximately 4 weeks apart. Study's 3 and 4 were administered to the same group of students with a 1-month interval. The age of all participating students ranged from 6 to 11. The demographic information for the students is presented in Table 2.

**Table 2:** Demographic characteristics of participants.

Study	Gender		Grade				Total
	Girls	Boys	Grade 1	Grade 2	Grade 3	Grade 4	
Study I	508	452	261	250	195	254	960
Study II	251	253	95	130	139	140	504
Study III	496	458	-	273	356	325	954
Study IV	115	103	-	68	74	81	223
Total							2,418

As shown in Table 2, first-grade students were not included in Study III (criterion-related validity) or Study IV (test–retest reliability), as these studies were conducted during the fall semester when first-grade students had only recently begun acquiring literacy skills. To minimize potential measurement bias associated with emerging reading abilities and self-report responses, these analyses were limited to students in Grades 2–4. In contrast, Studies I and II (PCA and CFA) were conducted during the spring semester, when first-grade students had already acquired sufficient literacy skills and were therefore included in the analyses.

### 2.4 Measures

#### 2.4.1 A personal information form

A personal information form was used to obtain information regarding the students' gender, age, nickname and grade.

#### 2.4.2 Soft skills scale for primary school students (SSS-S)

Soft Skills Scale for Primary School Students (SSS-S) final version is composed of 19 items grouped under six distinct factors: interpersonal relations, emotional control, responsibility, study skills, self-control, and empathy. It employs a three-point Likert format, where participants indicate their responses by choosing among “Always,” “Sometimes,” or “Never.”

#### 2.4.3 The social-emotional learning skills scale

The Social-Emotional Learning Skills Scale (SEL-SS) created by Esen-Aygun and Sahin-Taskin (2017), was designed to assess the social-emotional learning levels of primary school students. The scale consists of 27 items distributed across seven dimensions: relationships with peers, perception of friendship, persistence, achievement, self-management, impulse control, and self-confidence. Research findings confirm that the SEL-SS is both a reliable and valid tool for evaluating the social-emotional competencies of primary school children. The SEL-SS was selected as the criterion measure because social-emotional learning and soft skills are closely related and often used interchangeably in the literature. While the interpersonal skills and emotional control dimensions of soft skills substantially overlap with social-emotional learning competencies, the soft skills construct encompasses a broader range of competencies, including cognitive skills, which extend beyond the scope of SEL. Therefore, the SEL-SS was considered the most appropriate, as it measures a conceptually related but distinct construct. Accordingly, a moderate positive correlation between the two scales was theoretically expected.

### 2.5 Statistical analysis

The obtained data was screened, and any missing or erroneous entries were removed from the data set. The IBM SPSS software was used to analyze data. Prior to the analysis, the dataset was examined for missing values. Cases with substantial missing responses were excluded from the dataset. The normality of the data was assessed using skewness and kurtosis coefficients. All values fell within the acceptable range, indicating that the data approximated a normal distribution.

In Study I, Principal Component Analysis (PCA) was conducted to examine the underlying component structure of the SSS-S items. Prior to the analysis, the Kaiser–Meyer–Olkin (KMO) measure of sampling adequacy and Bartlett's test of sphericity were performed. The obtained values indicated that the data were suitable for PCA. The Varimax rotation technique was applied (Abdi, 2003). Although the dimensions of soft skills may theoretically be correlated, Varimax rotation was preferred because the primary objective of this phase was to obtain a simpler and more interpretable component structure for item reduction. Following the initial PCA, items with factor loadings below.40, substantial cross-loadings, or belonging to components with fewer than three items were removed. PCA was then repeated using the remaining items to obtain the final component structure.

In Study II, the LISREL software was used to analyze the data. Multiple fit indices that account for different aspects of the model were employed to evaluate the model's goodness of fit.

In Study III, the IBM SPSS software was used to analyze the correlation data, and the Jamovi software was used to calculate the Cronbach's alpha and McDonald's omega coefficients. Preliminary analyses were conducted to test normality and multicollinearity. A Pearson product–moment correlation coefficient was calculated to examine the relationship between the SSS-S and SEL-SS.

In Study IV, a Pearson product–moment correlation coefficient was computed to evaluate the test–retest reliability of the SSS-S. Moreover, a paired-samples *t*-test was conducted to determine whether there was any change in soft skills over a 1-month interval. Prior to Pearson correlation and independent-samples *t*-tests, the assumptions of normality and homogeneity of variances were evaluated. The normality assumption was supported by the skewness and kurtosis values, and Levene's test indicated that the assumption of homogeneity of variances was satisfied (*p* > 0.05).

### 2.6 Ethics approval

This study was reviewed and approved by Scientific Research and Publication Ethics Committee of Canakkale Onsekiz Mart University. Date of approval: 08.09.2023 Approval No: 11/49. The study protocol conformed to the ethical guidelines of the Declaration of Helsinki.

### 2.7 Informed consent

Legal permission was obtained from the National Education Directorate to implement the written Informed Consent form (Approval Date: 15.09.2023, Approval ID: E-60305806-44-84171284). Following legal permission, the written Informed Consent form was sent to the legal guardians of primary school students through the teachers to obtain their consent. The Informed Consent form was implemented between 12.02.2024 and 17.01.2025. Only those who brought back signed informed consent forms were included in the final data collection.

## 3 Results

An PCA was conducted in Study I. The cutoff value for the factor loadings was set at 0.40 (Tabachnick and Fidell, 2013). Items 1, 2, 7, and 11, whose factor loadings were below 0.40, were removed from the data set, and a second-order factor analysis was conducted.

The Kaiser–Meyer–Olkin (KMO) measure of sampling adequacy and Bartlett's Test of Sphericity were performed again in the second-order PCA. Accordingly, the KMO value was calculated as 0.81, and the Bartlett's test of sphericity result was significant (*p* < 0.001). Subsequently, the explained variance of the items was examined (see Table 3).

**Table 3:** Total variance explained by factors.

Component	Initial eigenvalues			^*^Extraction sums of squared loadings			Rotation sums of squared loadings		
	Total	% of Variance	Cumulative %	Total	% of Variance	Cumulative %	Total	% of Variance	Cumulative %
1	3.36	15.29	15.29	3.36	25.29	15.29	1.77	8.07	8.07
2	1.32	6.03	21.32	1.32	6.03	21.32	1.62	7.39	15.46
3	1.18	5.40	26.73	1.18	5.40	26.73	1.57	7.16	22.63
4	1.11	5.06	31.80	1.11	5.06	31.80	1.46	6.67	29.30
5	1.06	4.83	36.63	1.06	4.83	36.63	1.42	6.47	35.77
6	1.04	4.74	41.37	1.04	4.74	41.37	1.23	5.59	41.37
7	.98	4.46	45.83						
8	.97	4.42	50.26						
9	.93	4.27	54.53						
10	.91	4.13	58.66						
11	.90	4.09	62.75						
12	.88	4.02	66.78						
13	.86	3.92	70.70						
14	.83	3.80	74.50						
15	.81	3.71	78.22						
16	.80	3.64	81.87						
17	.72	3.31	85.18						
18	.71	3.23	88.42						
19	.69	3.15	91.57						
20	.64	2.94	94.52						
21	.61	2.79	97.30						
22	.59	2.69	100.00						

^*^Extraction method: principal component analysis.

**Figure 2:**
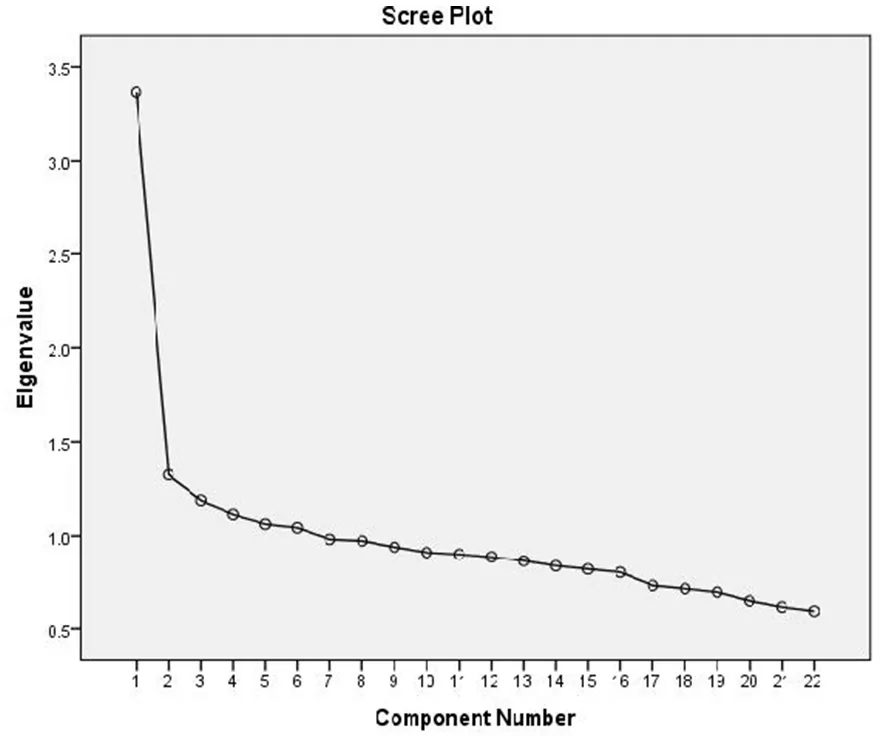
Scree plot.

As seen in Table 3, the SSS-S consists of a six-factor structure. Factor 1 explains 8.07% of the total variance, Factor 2 explains 15.46%, Factor 3 explains 22.63%, Factor 4 explains 29.30%, Factor 5 explains 35.77%, and Factor 6 explains 41.37% of the total variance. Additionally, a scree plot was examined to determine the number of factors (see Figure 2).

As seen in Figure 2, the breaking point occurs between five and six factors. Accordingly, it was understood that the six-factor structure obtained as a result of the PCA conducted within the scope of this study is consistent with the variance analysis results. The findings regarding the eigenvalues and communalities of the items were determined. Specifically, there were nineteen items with eigenvalues >1.00, and these items formed a distinct factorial structure. Principal Component Analysis (PCA) was employed during the initial stage of scale development to identify the underlying structure of the item pool and reduce the number of variables. PCA has also been widely used in educational and psychological scale development studies because it provides an efficient and stable initial solution, particularly in early exploratory analyses. The factors loading of the items are presented in Table 4.

**Table 4:** Factor loadings.

Item	1	2	3	4	5	6
	0.550					
**S5**	0.517					
**S21**	0.516					
**S22**	0.482					
**S19**		0.719				
**S23**		0.631				
**S17**		0.423				
**S12**			0.698			
**S6**			0.609			
**S20**			0.414			
**S13**				0.696		
**S14**				0.509		
**S4**				0.413		
**S8**					0.608	
**S16**					0.572	
**S15**					0.551	
**S10**						0.600
**S18**						0.524
**S3**						0.513

Extraction method: principal component analysis.

Rotation method: Varimax with Kaiser normalization.

As seen in Table 4, there are 19 items with factor loadings above 0.40 for the SSS-S, and these items form a six-factor structure. The factors obtained in the final step of the PCA were named based on the characteristics of the items.

In Study II, CFA was conducted. Following the PCA, the scale was determined to consist of 19 items and six factors. Although the items were measured using a three-point Likert-type scale, Maximum Likelihood estimation was employed because of the large sample size and its widespread use in psychometric validation studies.

This finding confirms the model established to determine the soft skill levels of primary school students. In addition to the path analysis results, the fit indices were examined to evaluate the validity and acceptability of the model. The goodness-of-fit indices were calculated as χ^2^(137) = 198.50, *p* = 0.00047, χ^2^/df = 1.45, RMSEA = 0.030, NFI = 0.801, TLI = 0.907, CFI = 0.926, SRMR = 0.041, GFI = 0.960, and AGFI = 0.944. Although the NFI value was generally below the recommended threshold, the remaining fit indices indicated that the proposed six-factor model provided an acceptable fit to the data. These indices suggest that the proposed model demonstrated an acceptable to good fit.

In Study III, criterion-related validity was examined. Subsequently, a Pearson product–moment correlation coefficient was computed to examine the relationship between the SSS-S and SEL-SS (see Table 5).

**Table 5:** Correlations between SSS-S and SEL-SS.

Scale		SSS-S	SEL-SS
SSS-S	Pearson correlation	1	0.601^**^
	Sig. (two-tailed)		0.000
	*n*	954	954
SEL-SS	Pearson Correlation	0.601^**^	1
	Sig. (two-tailed)	0.000	
	*n*	954	954

^**^Correlation is significant at the 0.01 level (two-tailed).

The moderate correlation coefficient shown in Table 5 reveals that although SSS-S and SEL-SS are related (*r* = 0.601, *p* < 0.001), they do not evaluate the same construct. This finding provides supporting evidence for the criterion-related validity of the SSS-S.

In Study III, the reliability coefficient of the scale was also calculated. Accordingly, internal consistency coefficients for the six subscales were as follows: interpersonal skills (α = 0.79*;* ω = 0.80), emotional control (α = 0.83; ω = 0.85), responsibility (α = 0.79; ω = 0.80), study skills (α = 0.78; ω = 0.80), self-control (α = 0.84; ω = 0.86), and empathy (α = 0.77; ω = 0.78), with an overall Cronbach's alpha of 0.79 and McDonald's omega of 0.80 for the total scale.

In Study IV, the participants completed the SSS-S twice at a 1-month interval. The mean SSS-S score in the first administration was 2.53, and the mean in the second administration was 2.59. The Pearson correlation coefficient between the two administrations was *r* = 0.834, indicating a high level of test–retest reliability (Table 6).

**Table 6:** Test-retest reliability.

Measure	*n*	Mean	sd	df	*t*	*p*
Test	223	2.5399	24,800	222	1.842	0.067
Retest	223	2.5903				
	***n***	**Correlation**				***p***
Test and retest	223	0.834^**^				< 0.001

Sig. *p* > 0.05, ^**^Correlation is significant at the 0.01.

As seen in Table 6, the results of the paired-samples *t*-test indicated that there were no significant differences in the mean SSS-S scores between the two time points (*t*(222) = 1.84, *p* > 0.05). Moreover, a Pearson product–moment correlation analysis revealed a strong positive association between the SSS-S scores from the first and second administrations (*r* = 0.83).

## 4 Discussion

In this study, conducted with 2,418 students from two different cities, we aimed to develop a scale for assessing the soft skills of primary school students and to examine its psychometric properties. The resulting instrument is intended to support educational stakeholders in assessing primary school students' soft skills. To achieve this aim, we conducted four separate studies and employed various statistical tests. Findings from the first of these studies, the PCA, indicated that the SSS-S has a structure consisting of six factors and 19 items. Although the initial conceptual framework adopted in this study consisted of three broad domains (interpersonal skills, emotional control, and cognitive skills), these domains were intentionally defined at a higher level of abstraction. During PCA, each broad domain differentiated into more specific but theoretically related dimensions. Specifically, interpersonal competence was represented by interpersonal skills and empathy; emotional competence by emotional control and self-control; and cognitive competence by study skills and responsibility. Thus, the six-factor solution does not represent a departure from the original conceptualization but rather a more refined representation of the multidimensional nature of soft skills in primary school students. This six-factor structure, which explains 41.37% of the total variance, indicates that a multifactor structure is appropriate for the SSS-S (Watkins, 2018).

The fit indices obtained from the CFA conducted in Study II indicated that the proposed six-factor model demonstrated an acceptable fit to the data, providing additional evidence for the structural validity of the SSS-S. The fit indices of the model were calculated as χ^2^/df = 997.63, RMSEA = 0.030, NFI= 0.801, TLI= 0.907, CFI = 0.926, SRMR = 0.0405, GFI = 0.960, and AGFI = 0.944. Taking together, these fit indices suggest that the proposed model demonstrated an acceptable to good fit to the data (Hooper et al., 2008; Schumacker and Lomax, 2010). The results obtained in Study's I and II indicate that the factorial structure of the SSS-S is consistent with the existing literature.

The primary school years represent an important period in a child's cognitive, social-emotional, and moral development (Kim et al., 2024; Osman, 2019; Sankalaite et al., 2023; Wigelsworth et al., 2022). During this period, children begin to construct an identity through the holistic support of their development (Kaplan and Flum, 2012; Maehler and Hernández-Torrano, 2025). Healthy identity formation also facilitates integration into society (Haag et al., 2022). The validity and reliability findings suggest that the SSS-S provides a structured assessment of soft skills that are associated with the holistic development and social integration of primary school students.

The first factor of the SSS-S is interpersonal skills. Primary school students' interpersonal skills, such as communication, cooperation, leadership, and conflict resolution, are an important component of soft skills. These skills enable students to establish qualified interactions with other individuals while also helping them find a place within the social structure. Interpersonal skills is a skill that can be developed through education (Cui, 2025). In this context, determining students' interpersonal skills levels is the first step in developing this skill. This interpersonal skills subdimension is conceptually consistent with previous measures of social competence and interpersonal functioning reported in the literature (Elliott and Gresham, 2008; Zhou and Ee, 2012). Another sub-dimension of the scale is empathy. Empathy, which is an important dimension of human interaction, is a soft skill that includes both cognitive and emotional components (Jardim et al., 2022). Supporting the development of empathy skills in children strengthens social awareness skills in terms of social-emotional development; promotes ethical and responsible behaviors in terms of moral development; supports emotional balance and adaptability in terms of personality development; and fosters cognitive flexibility in terms of cognitive development (Eisenberg, 2010; Nasaescu et al., 2023; Schonert-Reichl et al., 2012; Van Dongen, 2020). The empathy factor is also consistent with previous instruments developed for primary school students (Overgaauw et al., 2017; Reid et al., 2013).

The other two factors of the SSS are self-control and emotional control. For primary school students, self-control is a skill that enables them to manage their behaviors in accordance with rules and social expectations, representing an important component of daily life (Duckworth et al., 2014). Emotional control, on the other hand, is the ability to recognize emotions, express them appropriately, and regulate intense emotional reactions (Eisenberg and Spinrad, 2004). Emotional control, which is also a determinant in terms of empathy and interpersonal skills, is important for a student's classroom adjustment and academic stability (Denham, 2019; Li et al., 2024). While self-control involves the regulation of behaviors, emotional control refers to the regulation of emotions. Together, these two subfactors represent complementary aspects of the emotional and behavioral dimensions of primary school students' soft skills. When the self-control scales in the literature are examined, it is observed that they are generally based on teacher observation (Kendall and Wilcox, 1979; Rohrbeck et al., 1991) or are designed for upper age groups in primary education (Jin and Ahn, 2020; Manapat et al., 2021). Similarly, scales related to the emotion regulation dimension of emotional control are mostly based on caregiver reports (Shields and Cicchetti, 1997) or focus on emotional awareness and expression (Caiado et al., 2023; Kaloustian et al., 2025). In this context, these subdimensions overlap with similar scales in the literature; however, due to their target group and student self-report structure, they may provide a more comprehensive assessment. Thus, through these two factors, the emotional and behavioral regulation processes of primary school students are integrated within the same measurement framework.

Soft skills include not only emotional and behavioral skills but also cognitive processes. These cognitive processes indirectly support children's academic achievement (Pabst et al., 2022). In this context, two subfactors emerged in this scale that focus on measuring children's soft skills cognitively: study skills and responsibility. Study skills refer to students' cognitive behaviors such as attention, concentration, following instructions, and persistence in acquiring knowledge, managing the learning process, and achieving academic goals (Zimmerman, 2002). These skills are associated with more effective academic learning processes. The responsibility factor reflects students' awareness of academic and social responsibility, such as time management, cooperative work, and behaving in accordance with school rules (Martín-Antón et al., 2020). Responsibility is an important indicator of cognitive-emotional components such as learning discipline, perseverance, and self-regulation that develop during the primary school period. Since many scales in the literature are addressed under the framework of academic self-regulation (Ebbes et al., 2024), unlike many existing instruments, study skills and responsibility emerge as distinct subdimensions in the present scale. It is also understood that the limited number of existing scales do not focus on primary school students (Daisy and Nair, 2018; Fernández Río et al., 2019) or are directed toward responsibility within the context of a specific course (Açikgöz and Güler, 2021). When these two factors are considered together, they reflect not only students' cognitive competencies but also their capacity to adapt these competencies to the academic environment. Therefore, the study skills and responsibility dimensions included in the scale provide a structured framework for assessing the cognitive aspect of children's soft skills. When the factorial structure resulting from the PCA and CFA is examined, it is understood that the findings suggest that each subdimension represents a conceptually distinguishable aspect of primary school students' soft skills while contributing to the overall multidimensional structure of the scale.

To ensure the reliability of the SSS, criterion-related validity (CRV) was applied in Study III to determine its relationship with similar construct. Although there are existing scales focusing on various dimensions of primary school students' soft skills (Aridi et al., 2023; Colledani et al., 2024; Gülgez and Gündüz, 2019; Harmanci and Aytar, 2023; Jardim et al., 2022; Kırıcı and Ekşi, 2022; Soto et al., 2022), to the best of our knowledge, there is no scale that directly measures soft skills in the 6–12 age group. Social-emotional skills show similarities with soft skills in terms of interpersonal skills and emotional control dimensions. In this context, we examined the correlation between the two scales. The findings indicate that the responses of primary school students on both scales exhibited a moderate correlation. When testing criterion-related validity, the correlation between two constructs is expected to range between 0.30 and 0.70, whereas a value above 0.80 indicates redundancy between the constructs (Gignac and Szodorai, 2016). Accordingly, in the present study, it was found that the SSS-S developed in this research and the SEL-SS share a similar theoretical foundation, while the findings suggest that the SSS-S retains a distinct structure in measuring the soft skills of primary school students.

Finally, we conducted test-retest reliability analysis to assess the stability of SSS-S over time, both a paired-sample *t*-test and a test–retest correlation analysis were conducted in the Study IV. The paired-sample *t*-test indicated no significant difference between the mean scores obtained from the first and second administrations. Additionally, a high positive correlation was found between the two administrations. These findings support the temporal stability of the SSS-S, providing additional evidence for its test–retest reliability (DeVellis and Thorpe, 2021; Kline, 2016).

## 5 Conclusion

The findings obtained across the four studies provide evidence supporting the reliability and validity of the SSS-S for assessing the soft skills of primary school students. Taken together, the findings suggest that the proposed factor structure is conceptually coherent and supported by the psychometric evidence obtained in this study. The SSS-S may support evidence-based practices in schools by facilitating the assessment of students' soft skills. It may also inform future educational policies and intervention programs focused on soft skills. Overall, these findings suggest that the SSS-S has promising psychometric properties and may support school-based assessments conducted by school counselors as well as inform educational policymakers and curriculum developers in designing soft skills-oriented educational practices.

In literature, instruments commonly used to assess soft skills in primary school children tend to conceptualize social-emotional competence as a general and context-independent construct; they tend not to explicitly incorporate skills specific to the academic context as distinct dimensions and largely rely on psychometric evidence derived from adolescent samples. In contrast, the six-factor structure identified in the present study may offer a developmentally and culturally relevant framework for capturing the soft skill profiles of primary school students. In particular, the emergence of “studying skills” as an independent factor suggests that soft skills in this age group are closely intertwined with learning processes. Furthermore, the distinction between emotional control and behavioral self-control, together with the explicit inclusion of responsibility and studying skills, provides a context-sensitive assessment of children's soft skills within school settings.

### 5.1 Limitations

Although the study was conducted with a relatively large sample, the participants were drawn from only two cities. The generalizability of the findings is limited because the sample was taken from only two cities in Türkiye. Future researchers should validate the scale using more diverse samples from different regions of Türkiye and from different cultural and educational contexts. Furthermore, researchers wishing to use the scale in international comparative research are advised to first conduct cross-cultural validity and measurement invariance analyses.

Although PCA was employed as the initial exploratory technique for item reduction, the resulting six-component structure was subsequently supported through confirmatory factor analysis using an independent sample. Future studies may further examine the dimensional structure using common factor extraction methods.

The psychometric properties of this measurement tool are limited to primary school students. Researchers who wish to use the scale at the middle or high school levels are recommended to conduct new validity and reliability analyses. The criterion-related validity and test–retest studies were limited to second-, third-, and fourth-grade students. These tests were not administered for first-grade students, as they had only just begun learning to read and write. This study is limited to a sample from Turkey. Additionally, although the SSS-S was developed within the Turkish cultural and educational context, its conceptual foundation is based on internationally recognized frameworks of soft skills. However, cultural norms and educational systems can influence soft skills. Therefore, future studies should investigate the cross-cultural validity of the scale. This will contribute to establishing its universality and identifying culturally specific dimensions.

## Data Availability

Data are available from the corresponding author on request.
