# Correction: Development and psychometric validation of the soft skills scale for primary school students

**DOI:** 10.3389/fpsyg.2026.1967971

**Published:** 2026-09-17

**Authors:** Hanife Esen Aygün, Mehmet Aşıkcan, Serife Gonca Zeren

**Affiliations:** 1 Department of Primary School Education, Canakkale Onsekiz Mart University, Canakkale, Türkiye; 2 Department of Primary School Education, Necmettin Erbakan University, Konya, Türkiye; 3 Department of Guidance and Psychological Counseling, Canakkale Onsekiz Mart University, Canakkale, Türkiye

**Keywords:** development scale, primary school students, psychometric validation, school psychology, soft skills

An incorrect informed consent date was provided.

A correction has been made to section **2 Materials and Methods**, *sub-section 2.7 Informed consent*, paragraph 1:

“The Informed Consent form was implemented between 12.02.2024 and 17.01.2025.”

The Generative AI statement was erroneously given as “The author(s) declared that Generative AI was used in the creation of this manuscript.”

The correct Generative AI statement is:

